# Food intake, tumor growth, and weight loss in EP_2_ receptor subtype knockout mice bearing PGE_2_-producing tumors

**DOI:** 10.14814/phy2.12441

**Published:** 2015-07-21

**Authors:** Britt-Marie Iresjö, Wenhua Wang, Camilla Nilsberth, Marianne Andersson, Christina Lönnroth, Ulrika Smedh

**Affiliations:** 1 Surgical Metabolic Research Laboratory, Department of Surgery, Sahlgrenska Academy, University of GothenburgGothenburg, Sweden; 2 Department of Geriatric Medicine and Department of Clinical and Experimental Medicine, Linköping UniversityLinköping, Sweden

**Keywords:** Anorexia, cachexia, EP receptor, hypothalamus, microarray analysis, Prostaglandin D synthase

## Abstract

Previous studies in our laboratory have demonstrated that prostaglandin (PG) E_2_ is involved in anorexia/cachexia development in MCG 101 tumor-bearing mice. In the present study, we investigate the role of PGE receptor subtype EP_2_ in the development of anorexia after MCG 101 implantation in wild-type (EP_2_^+/+^) or EP_2_-receptor knockout (EP2^−/−^) mice. Our results showed that host absence of EP_2_ receptors attenuated tumor growth and development of anorexia in tumor-bearing EP_2_ knockout mice compared to tumor-bearing wild-type animals. Microarray profiling of the hypothalamus revealed a relative twofold change in expression of around 35 genes including mRNA transcripts coding for Phospholipase A_2_ and Prostaglandin D_2_ synthase (*Ptgds*) in EP_2_ receptor knockout mice compared to wild-type mice. Prostaglandin D_2_ synthase levels were increased significantly in EP_2_ receptor knockouts, suggesting that improved food intake may depend on altered balance of prostaglandin production in hypothalamus since PGE_2_ and PGD_2_ display opposing effects in feeding control.

## Introduction

Tumors are known to cause inflammation through release of cascades of inflammatory signals including interleukins and prostaglandins in order to promote growth (Lönnroth et al. 1995). Cytokines and eicosanoides cause a variety of secondary physiological responses of the host, including anorexia and weight loss (Plata-Salaman 1999; Furuyashiki and Narumiya 2011). The precise mechanisms of prostaglandins to alter feeding and metabolism are, however, not fully known. Prostaglandins act on specific EP receptor subtypes which are transmembrane spanning, G-protein coupled receptors classified as EP_1_, EP_2_, EP_3_, and EP_4_. Each EP receptor is associated with a unique G-protein and a second messenger system, but signaling can also be transduced by G-protein-independent mechanisms (Jiang and Dingledine 2013a). Previously, attention has been paid to the role of EP receptor subtypes 1, 3, and 4 in anorexia secondary to tumor growth (Wang et al. 2001, 2005a; Ruud et al. 2013). However, since cyclooxygenase inhibition by indomethacin failed to improve food intake but maintained body composition in tumor-bearing animals genetically depleted of EP_1_ or EP_3_ receptors, it would appear that these receptors do not participate in a prostaglandin-induced anorexic response (Wang et al. 2005a). A possible candidate for central anorexia could be EP_4_ receptor since ICV injection of an EP_4_ antagonist blocked the anorexic effect of PGE_2_ administration in healthy mice (Ohinata et al. 2006). However, PGE_2_-EP_4_ receptor ligand binding does not seem to be the underlying mechanism in tumor-induced hypophagia since CNS-specific disruption of EP_4_ receptors did not alter the anorexic response in MCG 101 tumor-bearing animals (Ruud et al. 2013).

From a clinical perspective, PGE_2_ has raised interest since it may be released from epithelial tumors such as colon cancer in progressive disease (Yang et al. 1998; Cahlin et al. 2008). There are several possible mechanisms for PGE_2_ to reach its central target receptors. PGE_2_ is highly lipophilic and can readily cross the blood–brain barrier but has a very short half-life in the circulation, and passive diffusion has been suggested to be of less importance (Ruud et al. 2013). Instead circulating PGE_2_ was suggested to act in the circumventricular organs and induce central PG synthesis and release via COX-activation (Laflamme et al. 1999). Prostaglandins display significant cross-reactivity on all of the four subtypes of EP receptors (Kiriyama et al. 1997) and EP_1–4_ receptors are present in hypothalamus and brainstem areas of relevance for feeding control and metabolism (Zhang and Rivest 1999; Wang et al. 2005b; Ruud et al. 2013).

The aim of the present study was to evaluate the role of subtype EP_2_ receptor signaling for development of anorexia in tumor-bearing animals since genetic knockout studies could not verify a role of other PGE receptor candidates as EP_1_, EP_3_, or EP_4,_ in mediating the prostaglandin-induced anorexic response of the tumor-bearing host (Wang et al. 2005a; Ruud et al. 2013). For this purpose we used a solid tumor model, MCG 101, which induces anorexia and cachexia in part due to elevated intrinsic production of PGE_2_. In order to explore the role of the EP_2_ receptor for anorexia development, an EP_2_^−/−^ knockout mice model was used.

## Materials and Methods

### Animal experiments

The animal experimental protocol was approved by the Regional committee for animal ethics in Göteborg. Adult, male and female and age-matched EP_2_^−/−^ and EP_2_^+/+^ mice (C57BL/6 genetic background) (Tilley et al. 1999) were bred and housed in plastic cages in a temperature controlled room with a 12 h dark/light cycle and received standard laboratory rodent chow (B & K Universal AB, Stockholm, Sweden). Animal groups were tumor-bearing (TB) and sham-treated controls (FF) in EP_2_^−/−^ and EP_2_^+/+^ mice. All animals had free access to tap water and food at all times before and during experiments. Prior to experiments, mice were transferred to cages with wire floor that permitted collection and quantification of spilled food by weighing. Daily food intake and body weight were registered in the morning between 08.00 and 09.00. Animals were allowed 3 days adaptation to wire floors before the start of experiments (day 0) (Lönnroth et al. 1995; Wang et al. 2005a,c). Tumor-bearing mice were implanted s.c. bilaterally in the flank with a 3–5 mm^3^ of a transplantable MCG-101 methylcholanthrene-induced tumor under general anesthesia (Isofluran, inhaled concentration 2.7%) (Lundholm et al. 1978). Control mice were sham implanted. All mice were sacrificed on day 10 upon tumor implantation between 8–11 am. Blood samples were obtained by cardiac puncture during general anesthesia for plasma PGE_2_ determination followed by 20 mL 4°C transcardiac saline perfusion (Lönnroth et al. 1995; Wang et al. 2001). The brains were rapidly removed and hypothalamus was dissected free, snap-frozen in liquid nitrogen, and kept at −80°C until micro-array analyses. Dry tumor weight, water content, fat-free carcass weight, and whole-body fat were determined as described (Eden et al. 1983).

### RNA extraction

Total RNA was extracted using RNeasy Lipid Tissue mini kit (Qiagen GmbH, Hilden, Germany) with on column DNase treatment included according to kit protocol. Quality of RNA was checked in an Agilent 2100 BioAnalyzer with the RNA 6000 Nano Assay kit (Agilent Technologies, Inc., Santa Clara, CA). The concentration of RNA was measured in a Nano Drop ND-1000A spectrophotometer (NanoDrop Technologies, Inc., Wilmington, DE). Hypothalamic mRNA for microarray analysis was pooled from seven mice in each group.

### Real-time PCR

Two hundred nanograms of total RNA from each hypothalamus were reverse transcribed in a cDNA synthesis reaction using oligo d(T) primers according to the manufacturer’s instructions (Advantage® RT for PCR kit; Takara Bio Europe/Clontech, Saint-Germain-en-Laye, France). Positive and negative controls were included in each run of cDNA synthesis. Predesigned primers from Qiagen were used for analysis of mouse *Ptgs1*, (Cox1, Assay 00155330) *Ptgs2*, (Cox2, Assay QT00165347) and *Ptgds* (PGD_2_ synthase, Assay QT00098049). Real-time PCR analysis was performed using either QantiTect SYBR Green kit or LightCykler FastStart DNA MasterPLUS SYBR Green I kit (Roche Diagnostics Scandinavia AB, Bromma, Sweden). Two microliter of diluted cDNA and 2 *μ*L of primer were used for each reaction of 20 *μ*L. All samples were analyzed in duplicates, and positive and negative results were included in each run. A LightCykler 1.5 instrument was used for all analyses. Quantitative results were produced by the relative standard curve method using GAPDH as housekeeping gene, which was equally expressed among groups.

### Microarray expression profiling

Five hundred nanograms of pooled total RNA from each group were labeled with Cyanine 3-dCTP or Cyanine 5-dCTP (GE Healtcare Life sciences, Uppsala, Sweden) in a cDNA synthesis reaction using the Agilent Fluorescent Direct Label Kit (*n* = 7/group). Whole Mouse Genome Oligo Microarray (4 × 44K; Agilent Technologies) containing 41,174 features, including positive and negative control spots, were used. Hybridization was performed during 18 h with EP_2_^−/−^ TB versus EP_2_^+/+^ TB cDNA in a dual-color experiment, followed by posthybridization washes according to “in situ Hybridization Kit Plus” (Agilent Technologies) instructions. Two technical replicates were done. The microarrays were dried with nitrogen gas in laminar flow and images were quantified on an Agilent G2565 AA microarray scanner. Fluorescence intensities were extracted using the Feature Extraction software program v9.1.3.1. (Agilent Technologies). Dye-normalized, outlier- and background-subtracted values were imported with the FE Plug-in (Agilent Technologies) into GeneSpring software program v 12.5 that was used for data analysis. Of the 41,232 features on the array, 18,851 features from pooled hypothalamus RNA were detected as present, with a signal ≥2.6 SD above background signal; 1747 entities remained after *t*-test against zero (*P* < 0.05). Fold changes 1.5 of Log2 transformed ratios were considered statistically significant in gene expression and used for further analyses in Gene Ontology search and pathway analysis. A fold change of 1.5 corresponds to a change in gene expression of 50% which has been reported to generate reproducible sets of altered genes when compared across microarray platforms (Patterson et al. 2006).

### Statistics

Results are presented as mean ± SE. Food intake and animal weight over time were compared by two-way ANOVA for repeated measures. End point variables (tumor weight, body composition, plasma PGE_2_ concentration and mRNA levels) were compared by one-way factorial ANOVA followed by Fisher PLSD, or *t*-test when appropriate. *P* ≤ 0.05 was considered statistically significant in two-tailed tests. Statview for Windows v. 5.0.1 was used for statistical calculations. Statistical evaluations of microarray analyses were done in Genespring 12.5 software as described in the Materials and Methods section.

## Results

### Food intake

**Figure 1:**
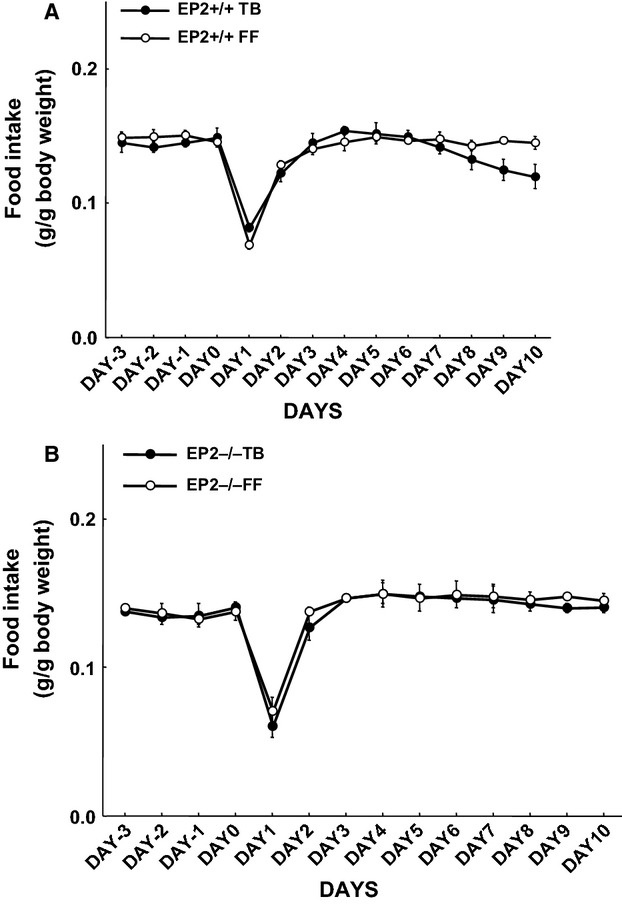
Time-course changes of food intake in EP_2_^+/+^ (A) and EP_2_^−/−^ (B) tumor-bearing mice (TB) and sham controls (FF). Food intake decreased significantly in TB wild-type mice from days 6 to 7 when tumor mass appeared (*P* < 0.05, A) (mean ± SEM, seven animals in each observation point; ANOVA for repeated measures).

Food intake declined significantly in wild-type tumor-bearing mice around day 7 and remained lower compared to sham controls in wild-type EP_2_^+/+^ mice (Fig.1A). There was no significant tumor-induced anorexia in tumor-bearing EP_2_^−/−^ knockouts (Fig.1B).

### Tumor weight and body composition

**Figure 2:**
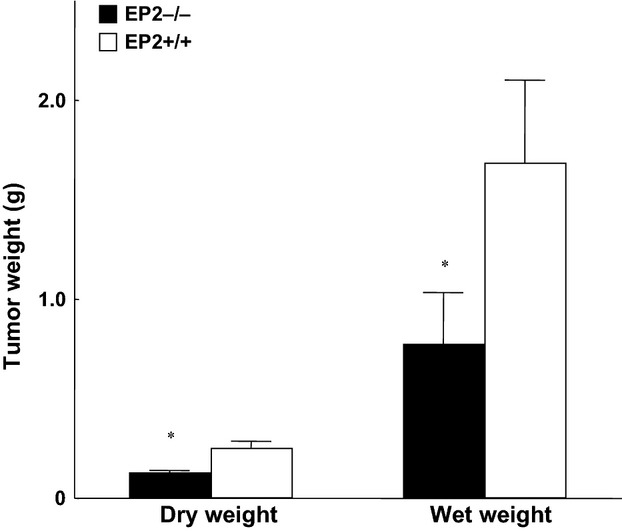
Tumor wet and dry weight at the end of experiments (day 10) in EP_2_^−/−^ and EP_2_^+/+^ tumor-bearing mice (mean ± SEM, *P < 0.05; seven animals in each group).

**Figure 3:**
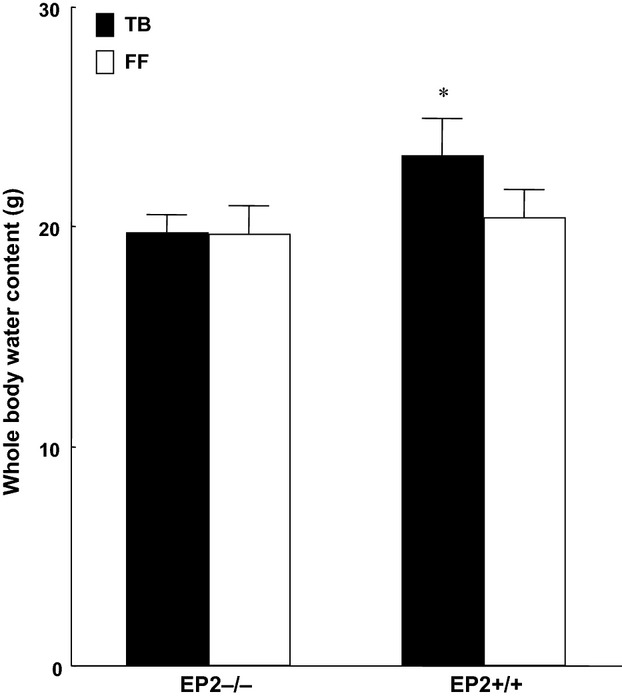
Whole-body water content in freely fed tumor-bearing mice (TB) and sham controls (FF) at the end of experiment (day 10) (mean ± SEM, *P < 0.05; seven animals in each group).

**Figure 4:**
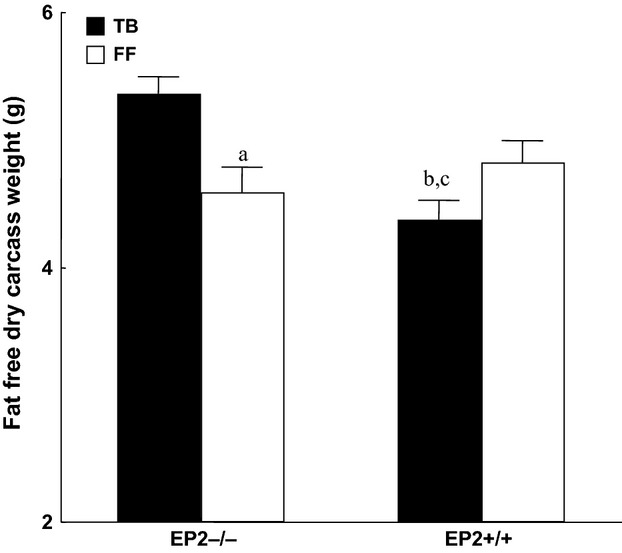
Whole-body fat-free carcass dry weight at the end of the experiments (day 10) in freely fed tumor-bearing animals (TB) and sham controls (FF) (mean ± SEM, (a) *P* < 0.01 versus TB EP_2_^−/−^ ; (b) *P* < 0.07 versus FF EP_2_^+/+^ ; (c) *P* < 0.001 versus TB EP_2_^−/−^; seven animals in each group).

**Figure 5:**
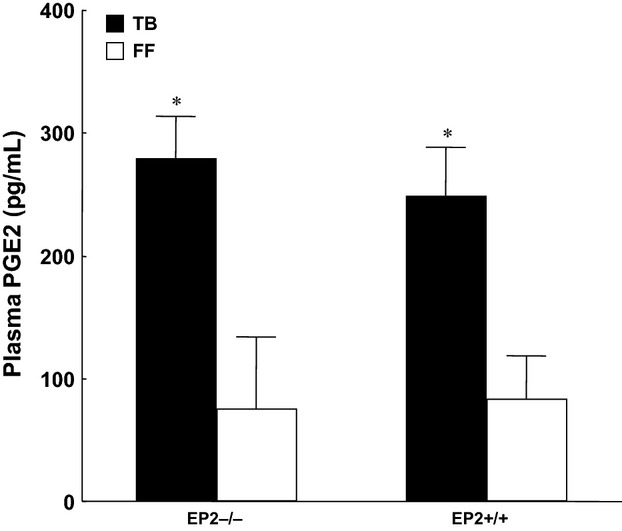
Plasma PGE_2_ concentration in tumor-bearing EP_2_^−/−^ and EP_2_^+/+^ mice compared to sham controls (FF) at the end of experiment (day 10) (mean ± SEM, *P < 0.01; seven animals in each group).

Tumor wet and dry weight were significantly lower at the end of the experiment in knockout mice (EP_2_^−/−^) compared to wild-type animals (EP_2_^+/+^) (*P* < 0.05; Fig.2). Pronounced alteration was observed in EP_2_^+/+^ groups due to larger tumors and whole-body water retention. Water retention did not occur in EP_2_^−/−^ mice (Fig.3). Fat-free carcass dry weight was significantly preserved in EP_2_^−/−^ tumor-bearing mice compared to wild-type EP_2_^+/+^ tumor-bearing mice (*P* < 0.001; Fig.4), while whole-body fat did not differ between EP_2_^−/−^ and wild-type tumor-bearing mice. Plasma PGE_2_ levels were similarly elevated in tumor-bearing mice compared to controls in both EP_2_^−/−^ and EP_2_^+/+^ mice (Fig.5).

### RNA expression in brain hypothalamus

Microarray analysis of pooled extracts of hypothalami from tumor-bearing EP_2_^−/−^ mice (*n* = 7) relative to tumor-bearing EP_2_^+/+^ animals (*n* = 7) showed differences in mRNA gene expression. We identified 182 genes that had above 1.5-fold change in relative expression between groups; 38 entities had above twofold difference (8 up- and 30 downregulated in EP2^−/−^ vs. EP2^+/+^ mice). The gene list with 1.5-fold changed genes was used for Pathway- and gene ontology analyses to find enriched pathways and categories of genes. Gene ontology search showed a significant match with the GO category GO:0048511 “Rhythmic processes” which contains genes involved in generation and maintenance of rhythms in the physiology of an organism. Additional significant matches were found with Wikipathways “IL2 signaling” *P* < 0.05 (two matching genes) and “TGFβ receptor signaling” *P* < 0.001 (five matching genes, Table1). We also found *Platg2f*, coding for Phospholipase A_2_ (down 2.2) and *Ptgds*; coding for Prostaglandin D_2_ synthase (up 2.2), among the genes with large change in expression between groups (TB EP2^+/+^ vs. TB EP2^−/−^).

**Table 1:** mRNA transcripts related to TGF-*β* signaling with altered levels in hypothalamic tissue from MCG 101 tumor-bearing EP_2_^−/−^ versus EP_2_^+/+^ mice at the end of experiment (day 10)

Gene name	Gene symbol	NCBI gene ID	Fold change	Regulation
SMAD family member 7	*Smad7*	17131	1.8	Up
Protein Kinase C, delta	*Prkcd*	18753	1.7	Up
Adaptor-related protein complex2, beta 1 subunit	*Ap2b1*	71770	1.6	Up
Mitogen-activated protein kinase kinase 6	*Map2k6*	26399	1.5	Up
Lympoid enhancer-binding protein	*Lef1*	16842	1.6	Down

**Figure 6:**
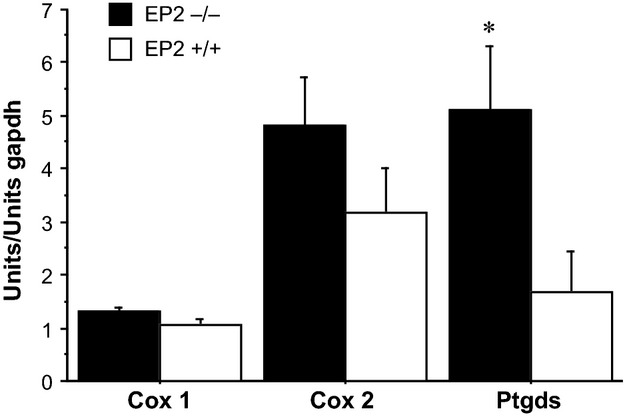
Levels of hypothalamic Cox-1, Cox-2, and Prostaglandin D_2_ synthase, mRNA in tumor-bearing EP_2_^−/−^ and EP_2_^+/+^ mice at the end of experiment (day 10) (mean ± SEM, *P < 0.05; seven animals in each group).

By real-time PCR we confirmed changes in *Ptgds* (Prostaglandin D_2_ synthase) and extended our analysis to include additional genes relevant for prostaglandin production *Ptgs*1 (Cox1), *Ptgs2* (Cox2) (*n* = 7/group). Hypothalamic Cox1 levels were significantly lower while Cox2 levels were increased in tumor-bearing mice compared to controls (Table2), while Cox2 expression was not significantly altered between tumor-bearing EP_2_^−/−^ and EP_2_^+/+^ mice (Fig.6). Prostaglandin D_2_ synthase was, however, significantly increased in EP_2_^−/−^ tumor-bearing mice compared to EP_2_^+/+^ tumor-bearing animals (Fig.6).

**Table 2:** Relative concentrations of Cox 1 and Cox 2 mRNA transcripts in hypothalamic tissue from EP_2_^−/−^ and EP_2_^+/+^ MCG 101 tumor-bearing mice (TB) and sham-implanted controls (FF) at the end of experiment (day 10, mean ± SEM)

	EP2^+/+^	EP2^−/−^
Cox 1		
TB	1.06 ± 0.11^a^	1.32 ± 0.08^a^
FF	2.24 ± 0.12	1.99 ± 0.07
Cox 2		
TB	3.17 ± 0.85^b^	4.82 ± 0.9^c^
FF	1.73 ± 0.21	1.39 ± 0.19

a *P* < 0.001 versus corresponding FF control of same genetic type.

b *P *< 0.15 versus corresponding FF control of same genetic type.

c *P* < 0.01 versus corresponding FF control of same genetic type.

## Discussion

In the present study, we examined the role of the EP_2_ receptor in anorexia development in mice carrying tumors that induce anorexia/cachexia and released increased levels of PGE_2_. We confirmed the results previously observed in the wild-type tumor-bearing groups, where exposure to MCG 101 over 10 days caused reductions of food intake and fat-free carcass weight (Cahlin et al. 2000; Wang et al. 2005a,c). We also found that host absence of EP_2_ receptors retarded MCG 101 tumor growth and maintained food intake and fat-free carcass weight. Genetic knockout of host EP_2_ receptors lead to significant changes in expression of mRNA transcripts related to prostanoid production in brain hypothalamus.

In the MCG 101 model the tumor cells produce prostaglandin E_2_, which consequently leads to elevated plasma levels of PGE_2_ (Lönnroth et al. 1995). Although prostaglandin E_2_ is suggested to cross the blood–brain barrier our previous study found no elevation of PGE_2_ or its metabolites in cerebrospinal fluid (Ruud et al. 2013). However, indomethacin treatment decreased anorexia concomitant with normalized plasma PGE_2_ levels (Wang et al. 2005a), suggesting COX dependency. We have previously suggested that anorexia is dependent on COX-1 expression rather than COX-2 in this model, since a COX-1 inhibitor delayed onset of anorexia while a selective COX-2 inhibitor was without such effect (Ruud et al. 2013). In the present experiments we found no change in relative expression of either COX-1 or COX-2 mRNA in hypothalamus from tumor-bearing EP_2_ receptor knockouts compared to tumor-bearing wild-type mice. However, both COX-1 and COX-2 mRNA expressions were significantly altered relative to sham-treated mice. Seen together, it appears that prostaglandins attenuate appetite and stimulate tumor growth which leads to overt cachexia. However, it remains to be determined whether systemic prostaglandins or brain PG production are of relevance. Likely, systemic PGE_2_ stimulates tumor growth while hypothalamic PGE_2_ production promotes anorexia. In earlier experiments we reported that loss of host EP_1_ or EP_3_ receptors did not alter anorexia in mice carrying MCG 101 tumors despite effects on tumor growth and body composition by indomethacin treatment (Wang et al. 2005a). Moreover, food intake was improved by short-term treatment by Cox-inhibitors without any effects on tumor size (Ruud et al. 2013). Such findings suggest separate effects of systemic and brain PG production and/or signaling linked to anorexia/cachexia secondary to tumor growth.

To identify other potential CNS mechanisms behind altered anorexia in EP_2_ receptor knockout mice we performed microarray analyses of hypothalamic extracts from tumor-bearing EP_2_^−/−^ mice relative to EP_2_^+/+^ animals. In total, there was a 1.5-fold change difference in expression of around 180 genes. A metabolic pathway search revealed possible involvement of TGF*β* signaling, which is associated with inflammatory response and reported to regulate COX/PGE_2_ levels, also in CNS (Luo et al. 1998; Minghetti et al. 1998; Matsumura et al. 2009; Fang et al. 2014), although our mice did not display altered COX mRNA levels., However, we found changed expression of other genes directly involved in PG production, such as increased amount of mRNA for Prostaglandin D_2_ synthase, and decreased expression of Phospholipase A_2_ from hypothalami of tumor-bearing EP_2_^−/−^ mice compared with EP_2_^+/+^ animals. Thus, reduced expression of Phospholipase A_2_ could reflect adaptation of PG production in the brain secondary to lack of EP_2_ receptors, contributing to improved food intake, although CNS levels of prostaglandins were not measured in present experiments.

PGD_2_ and PGE_2_ are positional isomers and have several opposing effects in physiological processes as sleep, body temperature, and feeding behavior (Kandasamy and Hunt 1990; Hayaishi 1991; Ohinata and Yoshikawa 2008). PGE_2_ and PGD_2_ are produced from the same precursor, PGH_2_, and is then converted to PGE_2_/PGD_2_ by specific enzymes. PGE_2_ is produced by the different isoforms of Prostaglandin E_2_ synthases whereas Prostaglandin D_2_ synthase produces PGD_2_. Recent findings report that central administration of PGD_2_ was associated with stimulation of food intake (Ohinata et al. 2008). Moreover, intraventricularly administered PGD_2_ was reported to stimulate food intake via DP_1_ receptor activation (Ohinata et al. 2008). The orexigenic effect of PGD_2_ was suggested to stimulate food intake via activation of NPY Y_1_ (Ohinata et al. 2008), the most orexigenic of the NPY receptors (Blomqvis and Herzog 1997), and increased mRNA levels of Prostaglandin D_2_ synthase were found in brain tissue of fasted mice as well as in food-restricted rats, without similar increases in tumor-bearing animals, supporting its role in appetite control (Ohinata et al. 2008; Pourtau et al. 2011). Therefore, it is plausible that maintained food intake in the EP_2_^−/−^ tumor mice was induced by increased DP_1_ receptor activity.

Our present and previous results suggest that host EP receptors are involved in control of tumor growth. In the present study, loss of host EP_2_ receptors reduced tumor growth which was also observed in our previous studies on EP_1_-deficient mice, whereas a lack of EP_3_ receptors increased tumor growth (Wang et al. 2005a). Earlier preclinical and clinical studies, suggest a role for cyclooxygenases and prostaglandins in tumor progression, although their downstream signaling is still not well understood. Our finding of reduced MCG 101 tumor growth agree with findings of reduced tumor growth in several other models, such as the syngenic colorectal cancer cell line MC26 as well as Lewis lung carcinoma in hosts lacking EP_2_ receptors (Yang et al. 2003). The importance of EP_2_ receptors for cancer cell proliferation has also been demonstrated using newly discovered selective EP_2_ antagonists (Jiang and Dingledine 2013b).

In conclusion, we demonstrate the importance of EP_2_ receptors for anorexia, cachexia progression in tumor-bearing mice, possibly mediated by altered balance of PGE_2_/PGD_2_ production in brain hypothalamus. Our results of reduced MCG 101 tumor growth are consistent with previous studies showing the importance of EP_2_ receptor signaling in tumor proliferation.
